# Mr. Shearsmith's Case of Incysted Tumor

**Published:** 1801-06

**Authors:** J. Shearsmith

**Affiliations:** Surgeon. Horsham, Sussex


### *To the Editors of the Medical and Phyfical Journal.*

Gentlemen,

If the following cafe be worth infertion in your valuable Journal, you will oblige me by letting it appear in your next number.

I am induced to think it is attended with a circumftance fcarcely, if at all, noticed by modern writers, (at leaft that have come within my own reading) and which I recollect only in Celfus.

Sarah Harding, ætat. 18, the daughter of a carpenter, at Warnham, near this place, confulted me for an encyfted tumor of the fteatomatous kind, fituated upon the left eye, and extending from the roots of the hair on the fuperior part of the forehead to the lower part of the eye-lid. She informed me it firft appeared when an infant of four months old, had gradually increafed to the prefent time, and although for many years it had not been attended with either pain or inconvenience, was now become troublefome, as well from its preffure upon the eye, as from the deformity occafioned by the depreffion of the eye-lid, which was half clofed by the weight of the tumor.

I gave her decidedly my opinion that nothing but extirpation would be of the leaft ufe; and having convinced her of the propriety of the operation, fhe confented to its being performed. I operated upon it in the prefence of Mr. Krazeifen, a gentleman from the York Hofpital, who is charged with the care care of the ſick in the barracks at this place, and who did me the favour to aſſiſt me in the operation.

On dividing the common integuments upon the ſac of the tumor, I found it adhering firmly at the bottom, and that in order to guard againſt a freſh accumulation of the ſame kind, it was neceſſary to diſſect it out through its whole extent. On puncturing the cyſt, I was aſtoniſhed to find half its contents to conſiſt of hair, in colour and length exactly correſponding with that on the eye-brow, partly looſe, and partly adhering to the bottom of the ſac. The loſs of blood in the operation, although pretty conſiderable, did not ſeem to call for the neceſſity of any ligature, as from the veſſels lying ſo immediately upon the os-frontis, compreſſion was all that was required; and having ſecured the wound by two ſtitches and ſlips of adheſive plaiſter, it healed very well by the firſt intention, and my patient is at this time in good health. In conſequence of a ſpaſmodic affection of the eye, I directed for her a draught for a few evenings, compoſed of Pulv. ipecac. comp. ℈j. and ſpt. ætheris. vitriolici. ʒſs. and, with an occaſional ſaline cathartic, was the only medicine I found neceſſary.

*Quere.*—In what way could the hair have originated? or is it an improbable conjecture that in the firſt formation of the eye-brow, part of it had been inverted? I apprehend there can be no doubt of the tumor being formed by the inflammation thoſe hairs had excited.

*Horsham, Sussex,* I am, &c.

*May* 4, 1801. J. SHEARSMITH, Surgeon.

